# Causal relationship between gut microbiota and cancers: a two-sample Mendelian randomisation study

**DOI:** 10.1186/s12916-023-02761-6

**Published:** 2023-02-21

**Authors:** Yiwen Long, Lanhua Tang, Yangying Zhou, Shushan Zhao, Hong Zhu

**Affiliations:** 1 Department of Oncology, Xiangya Hospital, Central South University, Changsha, Hunan 410008 People’s Republic of China; 2 National Clinical Research Center for Geriatric Disorders, Xiangya Hospital, Central South University, Changsha, Hunan 410008 People’s Republic of China; 3 Department of Orthopedics, Xiangya Hospital, Central South University, Changsha, Hunan 410008 People’s Republic of China

**Keywords:** Gut microbiota, Cancer, Mendelian randomisation, Genetics, SNPs

## Abstract

**Background:**

Evidence from observational studies and clinical trials suggests that the gut microbiota is associated with cancer. However, the causal association between gut microbiota and cancer remains to be determined.

**Methods:**

We first identified two sets of gut microbiota based on phylum, class, order, family, and genus level information, and cancer data were obtained from the IEU Open GWAS project. We then performed two-sample Mendelian randomisation (MR) to determine whether the gut microbiota is causally associated with eight cancer types. Furthermore, we performed a bi-directional MR analysis to examine the direction of the causal relations.

**Results:**

We identified 11 causal relationships between genetic liability in the gut microbiome and cancer, including those involving the genus *Bifidobacterium*. We found 17 strong associations between genetic liability in the gut microbiome and cancer. Moreover, we found 24 associations between genetic liability in the gut microbiome and cancer using multiple datasets.

**Conclusions:**

Our MR analysis revealed that the gut microbiota was causally associated with cancers and may be useful in providing new insights for further mechanistic and clinical studies of microbiota-mediated cancer.

**Supplementary Information:**

The online version contains supplementary material available at 10.1186/s12916-023-02761-6.

## Background

Gut microbiota residing in the gastrointestinal tract can be considered a potential environmental factor influencing human life. Currently, the gut microbiota has been implicated as a risk or preventive factor for a variety of diseases, including cancers, and is closely associated with the onset of colorectal cancer (CRC) [1]. Conversely, it has been pointed out that cancer also affects the gut microbiota in mice, which could induce gut microbiota disorders and cancer growth [2].

In contrast to observational studies, randomised controlled trials of gut microbiota could potentially help establish a causal relationship. Unfortunately, owing to the influence of objective factors, such as technology and research methods, the screening of strains involved in early diagnosis and prognosis still has great limitations. Consequently, most of the current research conclusions are based on observation of the composition and changes in the gut microbiota in patients’ faeces and on the results of trials in which gut microbiota were transplanted into gnotobiotic mice, which are influenced by a variety of factors, such as diet and antibiotic use [3–6]. In summary, whether the associations between the gut microbiota and cancers are causal and the direction of the causal associations are still unknown. It is thus important to explore the causal relationship between the gut microbiota and cancers.

Genome-wide association studies (GWASs) have tested millions of genetic variants across the genomes of many individuals to identify genotype–phenotype associations and have revolutionised the field of complex disease genetics over the past decade [7]. GWASs provide an agnostic approach for investigating the genetic basis of complex diseases. As of October 2022, the GWAS Catalog contained 6041 publications and 427,870 associations. In oncology, over 450 genetic variants associated with increased risks of common cancers have been identified. The clinical application of GWAS data has been providing opportunities for cancer prevention [8].

Mendelian randomisation (MR) analysis exploits the inherent properties of common genetic variations for a modifiable environmental exposure of interest and has become a widely used approach to explore the potential causal relationships between environmental exposures and diseases [9–11]. Two-sample MR analysis can utilise single-nucleotide polymorphism (SNP)-exposure and SNP-outcome associations from independent GWASs and combine them into a single causal estimate. As the number of GWASs on gut microbiota and diseases has increased rapidly [12, 13], large-scale summary statistics have become more widely available, allowing for two-sample MR analysis with significantly improved statistical power.

In the present study, we investigated the causal relationship between gut microbiota and a broad range of cancers by conducting a comprehensive two-sample MR analysis of eight cancers derived from the IEU Open GWAS project, including breast cancer, colorectal cancer, ovarian cancer, head and neck cancer, lung cancer, endometrial cancer, and prostate cancer. By applying a bi-directional MR approach, we can explore whether gut microbiota casually affects cancer risk and we can also examine whether the genetic predisposition to cancer risk causally influences the gut microbiota. Based on these, we tried to clarify the role of the gut microbiota in cancer development to eventually help to develop new treatment strategies, such as probiotic therapy, dietary modulations, and faecal microbiota transplantation (FMT) [14].

## Methods

### Exposure data

SNPs related to the human gut microbiome composition were selected as instrumental variables (IVs) from a GWAS dataset of the international consortium MiBioGen [13]. This was a multi-ethnic large-scale GWAS that coordinated 16S ribosomal RNA gene sequencing profiles and genotyping data from 18,340 participants from 24 cohorts from the USA, Canada, Israel, South Korea, Germany, Denmark, the Netherlands, Belgium, Sweden, Finland, and the UK to explore the association between autosomal human genetic variants and the gut microbiome. A total of 211 taxa (131 genera, 35 families, 20 orders, 16 classes, and 9 phyla) were included.

### Outcome data

We downloaded all traits reported in the IEU Open GWAS project https://gwas.mrcieu.ac.uk/ (updated to 2022.04.06, *N* = 40,427) and derived all cancer-related GWAS summary-level data. After screening the dataset and excluding duplicate studies, non-malignant tumours, and non-European ancestry, the GWAS summary-level data for the associations between genetic variants and cancers included those from the UK Biobank [15], the International Lung Cancer Consortium (ILCCO) [16, 17], the Prostate Cancer Association Group to Investigate Cancer Associated Alterations in the Genome (PRACTICA-L) consortium [18], the Medical Research Council-Integrative Epidemiology Unit (MRC-IEU) [19], the Ovarian Cancer Association Consortium (OCAC) [20], the Oncoarray oral cavity and oropharyngeal cancer [21], the Breast Cancer Association Consortium (BCAC) [22], FINNGEN [23], and Neale Lab (http://www.nealelab.is/uk-biobank/). Detailed information is provided in Additional file 1: Table S1.

### Instrumental variable selection

**Fig. 1:**
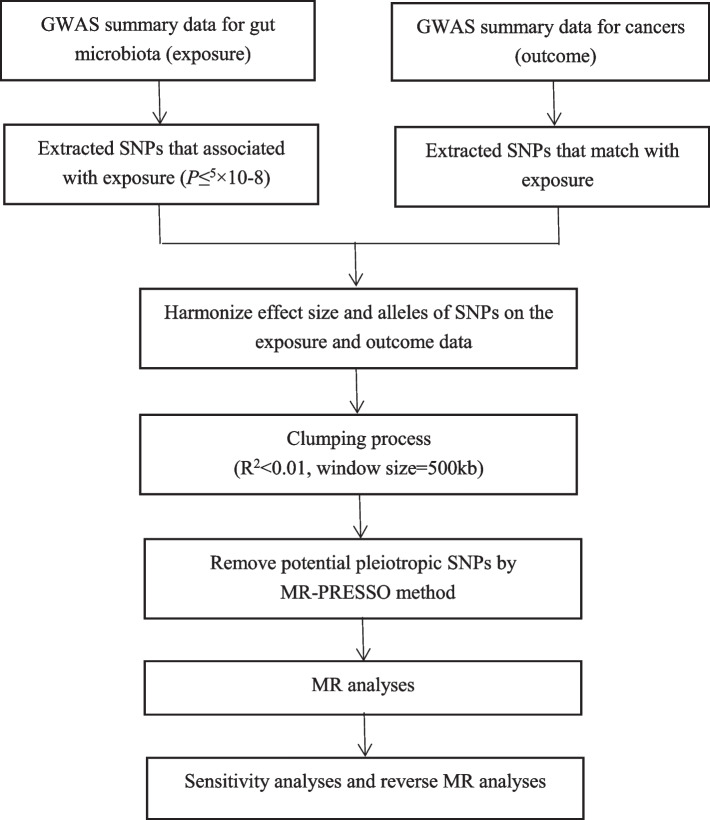
Study design and workflow

The flowchart of the study is presented in Fig. 1. Briefly, the gut microbiota served as the exposure, whereas cancer served as the outcome.

Bacterial taxa were analysed at five levels (phylum, class, order, family, and genus), and a distinct taxon was defined as a feature. To ensure the authenticity and accuracy of the conclusions on the causal link between the gut microbiome and cancer risk, the following quality control steps were used to select the optimal IVs. First, SNPs that were significantly related to the gut microbiome were selected as the IVs. Two thresholds were used to select the IVs. The first threshold selected SNPs less than the genome-wide statistical significance threshold (5 × 10^−8^) to serve as IVs. Unfortunately, after we selected SNPs, only a small number of gut microbiota were selected as IVs, and to explore more relations between cancers and gut microbiota to obtain more comprehensive results, we used the second threshold that identified SNPs that were smaller than the locus-wide significance level (1 × 10^−6^) and selected them as the second IVs set to find more potential causal associations. Second, the minor allele frequency (MAF) threshold of the variants of interest was 0.01. Third, one of the principles of the MR approach is that there is no linkage disequilibrium (LD) among the included IVs, as the presence of strong LD might result in biased results. In the current study, the clumping process (*R*^2^ < 0.01 and clumping distance = 10,000 kb) was conducted to assess the LD between the included SNPs. Fourth, an important step in MR is to ensure that the effects of the SNPs on the exposure correspond to the same allele as the effects on the outcome. To avoid distortion of strand orientation or allele coding, we deleted palindromic SNPs (e.g. with A/T or G/C alleles). During the harmonisation process, we aligned the alleles to the human genome reference sequence (build 37) and removed ambiguous and duplicated SNPs.

We applied MR-PRESSO and MR-Egger regression tests to monitor the potential horizontal pleiotropy effect. For each SNP, the MR-PRESSO outlier test calculated a *p*-value for its pleiotropy significance, whereas the MR-PRESSO global test calculated a *p*-value for overall horizontal pleiotropy. SNPs were sorted in ascending order in terms of their MR-PRESSO outlier test *p*-values and were then removed one by one. The MR-PRESSO global test was performed on the remaining SNPs each time an SNP was removed from the list. Recursion was repeated until the *p*-value for the global test was insignificant (*p* > 0.05). The list of SNPs remaining after removing pleiotropic SNPs was used for the subsequent MR analysis.

### MR analysis

We performed an MR analysis to investigate the causal relationship between microbiome features and common cancers. For features containing only one IV, the Wald ratio test was used to estimate the association between the identified IV and each cancer [24]. Five popular MR methods were used for features containing multiple IVs: inverse-variance weighted (IVW) test [25], weighted mode [26], MR-Egger regression [27], weighted median estimator (WME) [28], and MR-PRESSO [29]. The IVW method is reported to be slightly more powerful than the others under certain conditions [28]; therefore, the results with more than one IV were mainly based on the IVW method, with the other four methods serving as complements.

Additionally, we established a multiple testing significance threshold at each feature level (phylum, class, order, family, and genus), defined as *p* < 0.05/*n* (where *n* is the effective number of independent bacterial taxa at the corresponding taxonomic level). To assess the robustness of the results, several sensitivity analyses were performed. Leave-one-out analysis was performed to determine whether the causal signal was driven by a single SNP. This approach compares the variance explained by the IVs for both the exposure and outcome. If the IVs explain a greater variance in the exposure than that in the outcome, then the identified causal association could be considered directionally credible [30]. Furthermore, we calculated *F* statistics to evaluate weak instrument bias [31]. An *F*-value less than 10 indicated a weak instrument and was excluded.

All statistical analyses were performed using the R packages: two-sample MR [17] and MR-PRESSO [29].

### Heterogeneity

We performed a test for heterogeneity using Cochran’s *Q* statistics and the two-sample MR package between instruments. A *Q* larger than the number of instruments minus one provides evidence for heterogeneity and invalid instruments, or *Q* statistics significant at a *p-*value < 0.05 can imply the presence of heterogeneity [32, 33].

### Reverse MR analysis

To explore whether cancers have any causal impact on the identified significant bacterial genera, we also performed a reverse MR analysis (i.e. cancers as the exposure and the identified causal bacterial genus as the outcome) using SNPs that are associated with cancers as IVs. We used the MR Steiger directionality test [30] to examine whether exposure was directionally causal for the outcome.

## Results

### SNP selection

First, we identified 91, 307, 289, 310, and 397 SNPs associated with gut microbiota at the phylum, class, order, family, and genus levels, respectively, at a significance level of *p* < 5 × 10^−8^. We identified 228, 499,488, 811, and 1374 SNPs at the phylum, class, order, family, and genus levels, respectively, at a significance level of *p* < 1 × 10^−6^. After a series of quality control steps, 277 (genome-wide statistical significance threshold, *p* < 1 × 10^−6^) and 23 (genome-wide statistical significance threshold, *p* < 5 × 10^−8^) SNPs were selected as IVs.

The *F* statistics of the IVs were all largely > 10 (Additional file 2: Table. S2), indicating no evidence of weak instrument bias, and no evidence of pleiotropic effects was detected by the MR-PRESSO global test (*p* > 0.05). Eventually, after removing pleiotropic SNPs identified by the MR-PRESSO outlier test and the MR-Egger regression, there was no evidence of horizontal pleiotropy of the IVs (both MR-PRESSO global test *p* > 0.05 and MR-Egger regression *p* > 0.05).

Bacterial genera containing multiple SNPs were tested using the four MR methods to consider multiple testing corrections. In the set of SNPs less than the genome-wide statistical significance threshold (5 × 10^−8^) that served as IVs, the significance threshold for various taxa levels was set to the following: phylum *p* = 5 × 10^−2^ (0.05/1), class *p* = 5 × 10^−2^ (0.05/1), order *p* = 2.5 × 10^−2^ (0.05/2), family *p* = 1.25 × 10^−2^ (0.05/4), and genus *p* = 4.54 × 10^−3^ (0.05/11). In the set of SNPs less than the genome-wide statistical significance threshold (1 × 10^−6^) that served as IVs, the significance threshold for various taxa levels was set to the following: phylum *p* = 5.55 × 10^−3^ (0.05/9), class *p* = 3.33 × 10^−3^ (0.05/15), order *p* = 2.5 × 10^−2^ (0.05/15), family *p* = 2.08 × 10^−3^ (0.05/24), and genus *p* = 6.25 × 10^−4^ (0.05/80).

### Causal effects of gut microbiota on the development of eight cancer types

#### Breast cancer

**Fig. 2:**
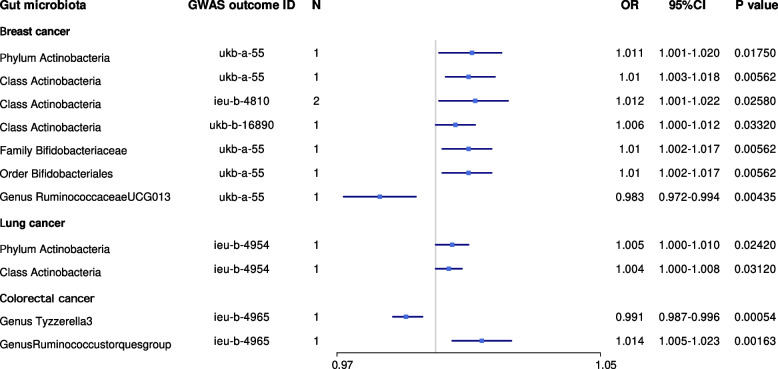
Mendelian randomisation results of causal effects between gut microbiome and cancer risk (*p* < 5 × 10^−8^)

In the set of IVs (*p* < 5 × 10^−8^), we found that the phylum *Actinobacteria *(odds ratio (OR) = 1.011, 95% CI = 1.001–1.020, *p* = 1.75 × 10^−2^, Wald ratio) was causally associated with breast cancer, and class *Actinobacteria* was causally associated with patients with breast cancer; the causal association between class *Actinobacteria* and breast cancer was identified in the Neale Lab (OR = 1.010, 95% CI = 1.003–1.018, *p* = 5.62 × 10^−3^, Wald ratio), UK Biobank (OR = 1.012, 95% CI = 1.001–1.022, *p* = 2.58 × 10^−2^, IVW), and MRC-IEU (OR = 1.006, 95% CI = 1.000–1.012, *p* = 3.32 × 10^−2^, Wald ratio). In addition, the genus *Ruminococcaceae* UCG013 was also causally associated with breast cancer (OR = 0.983, 95% CI = 0.972–0.994, *p* = 4.35 × 10^−3^, Wald ratio). Surprisingly, the family *Bifidobacteriaceae* and order *Bifidobacteriales* were also causally associated with breast cancer (OR = 1.010, 95% CI = 1.002–1.017, *p* = 5.62 × 10^−3^, Wald ratio); therefore, we performed an MR analysis in the UK Biobank database, which showed a similar result (OR = 1.009, 95% CI = 1.000–1.018, *p* = 3.57 × 10^−2^, Wald ratio) (Table 1, Fig. 2).

**Table 1:** Mendelian randomisation (MR) results of causal effects between gut microbiome and cancer risk (*P* < 5×10^-8^)

Gut microbiota (exposure)	Cancer type (outcome)	Consortium	Method	Number of SNPs	***β***	SE	***p-value***	OR	95% CI	Correct causal direction	Steiger ***p-***value
Phylum Actinobacteria	Breast cancer	Neale Lab	Wald ratio	1	1.11×10^−2^	4.66×10^−3^	1.75×10^−2^	1.01	1.001–1.020	True	6.36×10^−24^
Class Actinobacteria	Breast cancer	Neale Lab	Wald ratio	1	1.05×10^−2^	3.78×10^−3^	5.62×10^−3^	1.01	1.003–1.018	True	1.64×10^−33^
Class Actinobacteria	Breast cancer	UK Biobank	IVW	2	1.21×10^−2^	5.41×10^−3^	2.58×10^−2^	1.01	1.001–1.022	True	2.09×10^−17^
Class Actinobacteria	Breast cancer	MRC-IEU	Wald ratio	1	6.55×10^−3^	3.08×10^−3^	3.32×10^−2^	1.01	1.000–1.012	True	3.04×10^−17^
Family Bifidobacteriaceae	Breast cancer	Neale Lab	Wald ratio	1	9.97×10^−3^	3.60×10^−3^	5.62×10^−3^	1.01	1.002–1.017	True	7.79×10^−46^
Order Bifidobacteriales	Breast cancer	Neale Lab	Wald ratio	1	9.97×10^−3^	3.60×10^−3^	5.62×10^−3^	1.01	1.002–1.017	True	7.79×10^−46^
Genus *Ruminococcaceae* UCG013	Breast cancer	Neale Lab	Wald ratio	1	−1.63×10^−2^	5.70×10^−3^	4.35×10^−3^	0.98	0.972–0.994	True	1.12×10^−14^
Phylum.Actinobacteria	Lung cancer	UK Biobank	Wald ratio	1	5.49×10^−3^	2.44×10^−3^	2.42×10^−2^	1.01	1.000–1.010	True	1.06×10^−12^
Class Actinobacteria	Lung cancer	UK Biobank	Wald ratio	1	4.23×10^−3^	1.97×10^−3^	3.12×10^−2^	1.00	1.000–1.008	True	7.06×10^−18^
Genus *Tyzzerella3*	Lung adenocarcinoma	FINNGEN	Wald ratio	1	1.50	0.51	3.43×10^−3^	4.49	1.641–12.263	True	3.12×10^−4^
Genus *Tyzzerella3*	Colorectal cancer	UK Biobank	Wald ratio	1	−8.05×10^−3^	2.33×10^−3^	5.43×10^−4^	0.99	0.987–0.996	True	2.95×10^−7^
Genus *Ruminococcustorquesgroup*	Colorectal cancer	UK Biobank	Wald ratio	1	1.44×10^−2^	4.58×10^−3^	1.63×10^−3^	1.01	1.005–1.023	True	1.01×10^−6^
Genus *Ruminococcustorquesgroup*	Prostate cancer	FINNGEN	Wald ratio	1	−1.05	0.37	4.21×10^−3^	0.35	0.171–0.718	True	5.24×10^−7^
Family Peptostreptococcaceae	Gastric cancer	FINNGEN	Wald ratio	1	2.53	0.92	6.19×10^−3^	12.52	2.049–76.43	True	4.21×10^−6^
Order Gastranaerophilales	Oropharyngeal cancer	Oncoarray oral cavity and oropharyngeal cancer	Wald ratio	1	−1.28	0.55	1.92×10^−2^	0.28	0.094–0.811	True	2.23×10^−7^
Phylum Actinobacteria	Oral cavity cancer	Oncoarray oral cavity and oropharyngeal cancer	Wald ratio	1	−3.17	1.42	2.53×10^−2^	0.04	0.002–0.676	True	1.52×10^−7^
Class Actinobacteria	Oral cavity cancer	Oncoarray oral cavity and oropharyngeal cancer	Wald ratio	1	−2.03	0.99	3.99×10^−2^	0.13	0.019–0.910	True	2.01×10^−18^

*OR* Odds ratio, *SE* Standard error, *CI* Confidence interval

**Fig. 3:**
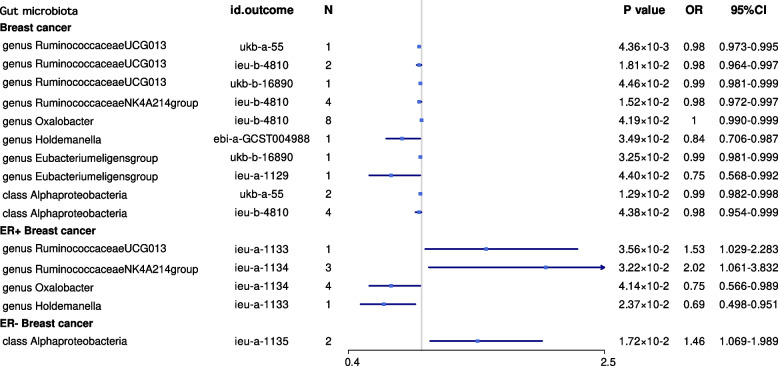
Mendelian randomisation results of causal effects between gut microbiome and breast cancer risk (*p* < 1 × 10^−6^)

We identified more gut microbiota related to breast cancer in the other set of IVs (*p* < 1 × 10^−6^), and we also found that the genus *Ruminococcus gnavus* was causally associated with breast cancer (OR = 1.466, 95% CI = 1.251–1.718, *p* = 2.15×10^−6^, IVW), especially ER^+^ breast cancer (OR = 1.549, 95% CI = 1.285–1.866, *p* = 4.27 × 10^−6^, IVW); the genus *Oscillibacter* was causally associated with ER− breast cancer (OR = 2.045, 95% CI = 1.393–3.002, *p* = 2.58 × 10^−4^, IVW) (Table 2, Fig. 3).

**Table 2:** MR results of causal effects between gut microbiome and cancer risk (*p* < 1×10^−6^)

Gut microbiota (exposure)	Cancer type (outcome)	Consortium	Method	Number of SNPs	***β***	SE	***p-value***	OR	95% CI	Correct causal direction	Steiger ***p-***value
Genus *Ruminococcusgnavusgroup*	Breast cancer	BCAC	IVW	2	0.38	8.08×10^−2^	2.16×10^−6^	1.47	1.25–1.73	True	4.13×10^−7^
Genus *Ruminococcusgnavusgroup*	ER+ breast cancer	BCAC	IVW	2	0.44	9.52×10^−2^	4.27×10^−6^	1.55	1.29–1.87	True	1.57×10^−6^
Genus *Oscillibacter*	ER− breast cancer	BCAC	IVW	2	0.72	0.20	2.59×10^−4^	2.05	1.39–3.00	True	1.17×10^−5^
Class Gammaproteobacteria	Endometrial cancer	MRC-IEU	Wald ratio	1	−6.38×10^−3^	1.91×10^−3^	8.44×10^−4^	0.99	0.99–1.00	True	2.22×10^−5^
Genus *Ruminococcus1*	Head and neck cancer	UK Biobank	Wald ratio	1	8.69×10^−3^	2.34×10^−3^	1.98×10^−4^	1.01	1.00–1.01	True	9.17×10^−12^
Genus *Ruminococcus1*	Oral and oropharyngeal cancer	UK Biobank	Wald ratio	1	7.25×10^−3^	2.04×10^−3^	3.71×10^−4^	1.01	1.00–1.01	True	8.76×10^−12^
Genus *Ruminiclostridium6*	Serous ovarian cancer: low malignant potential	OCAC	Wald ratio	1	2.47	0.67	2.33×10^−4^	11.87	3.18–44.33	True	2.83×10^−9^
Order Lactobacillales	Squamous cell lung cancer	ILCCO	IVW	2	1.16	0.38	2.17×10^−3^	3.18	1.52–6.67	True	8.74×10^−6^
Order Burkholderiales	Lung cancer	ILCCO	IVW	3	−0.59	0.20	2.75×10^−3^	0.55	0.38–0.82	True	7.49×10^−9^
Order Lactobacillales	Small cell Lung cancer	FINNGEN	IVW	2	4.23	1.43	3.06×10^−3^	68.83	4.18–1132.80	True	6.08×10^−6^
Genus *Terrisporobacter*	Prostate cancer	FINNGEN	Wald ratio	1	−0.96	0.24	6.79×10^−5^	0.38	0.24–0.61	True	4.56×10^−9^
Genus *Roseburia*	Prostate cancer	PRACTICA-L	Wald ratio	1	0.55	0.15	3.89×10^−4^	1.73	1.28–2.34	True	3.08×10^−13^
Class Verrucomicrobiae	Prostate cancer	UK Biobank	Wald ratio	1	−3.66×10^−2^	1.10×10^−2^	8.73×10^−4^	0.96	0.94–0.99	True	1.01×10^−4^
Family Verrucomicrobiaceae	Prostate cancer	UK Biobank	Wald ratio	1	−3.66×10^−2^	1.10×10^−2^	8.73×10^−4^	0.96	0.94–0.99	True	1.01×10^−4^
Order Verrucomicrobiales	Prostate cancer	UK Biobank	Wald ratio	1	−3.66×10^−2^	1.10×10^−2^	8.73×10^−4^	0.96	0.94–0.99	True	1.01×10^−4^
Class Alphaproteobacteria	Prostate cancer	PRACTICA-L	IVW	2	0.25	7.90×10^−2^	1.29×10^−3^	1.29	1.10–1.51	True	7.72×10^−11^
Order Verrucomicrobiales	Colorectal cancer	UK Biobank	Wald ratio	1	1.32×10^−2^	4.27×10^−3^	1.98×10^−3^	1.01	1.00–1.02	True	1.80×10^−5^
Order Desulfovibrionales	Colorectal cancer	UK Biobank	Wald ratio	1	1.53×10^−2^	5.15×10^−3^	2.98×10^−3^	1.02	1.00–1.03	True	6.97×10^−6^
Family Verrucomicrobiaceae	Colorectal cancer	UK Biobank	Wald ratio	1	1.32×10^−2^	4.27×10^−3^	1.98×10^−3^	1.01	1.00–1.02	True	1.80×10^−5^
Class Verrucomicrobiae	Colorectal cancer	UK Biobank	Wald ratio	1	1.32×10^−2^	4.27×10^−3^	1.98×10^−3^	1.01	1.00–1.02	True	1.80×10^−5^
Class Deltaproteobacteria	Colorectal cancer	UK Biobank	Wald ratio	1	1.55×10^−2^	5.15×10^−3^	2.98×10^−3^	1.02	1.02–1.03	True	9.00×10^−6^

#### Lung cancer

In the set of IVs (*p* < 5 × 10^−8^), we found that the phylum *Actinobacteria* (OR = 1.005, 95% CI = 1.000–1.010, *p* = 2.42 × 10^−2^, Wald ratio) and class *Actinobacteria* (OR = 1.004, 95% CI = 1.000–1.008, *p* = 3.12 × 10^−2^, Wald ratio) were causally associated with lung cancer. The genus *Tyzzerella3* was causally associated with lung adenocarcinoma (OR = 4.486, 95% CI = 1.641–12.263, *p* = 3.43 × 10^−3^, Wald ratio) (Table 1, Fig. 2).

**Fig. 4:**
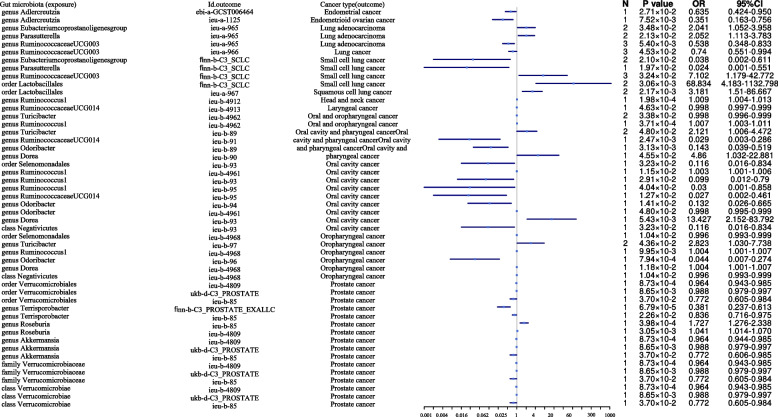
Mendelian randomisation results of causal effects between gut microbiome and other cancer risks (*p* < 1 × 10^−6^)

We identified more gut microbiota related to lung cancer in the other set of IVs (*p* < 1 × 10^−6^), and we found that the order *Lactobacillales* was causally associated with squamous cell lung cancer (OR = 3.181, 95% CI = 1.517–6.666, *p* = 2.17 × 10^−3^, IVW) and small cell lung cancer (OR = 68.83, 95% CI = 4.182–1132.79, *p* = 3.06 × 10^−3^, IVW), while the order *Burkholderiales* (OR = 0.553, 95% CI = 0.375–0.815, *p* = 2.75 × 10^−3^, IVW) was causally associated with lung cancer (Table 2, Fig. 4).

#### Colorectal cancer

In the set of IVs (*p* < 5 × 10^−8^), we found that the genus *Tyzzerella3* (OR = 0.991, 95% CI = 0.987–0.996, *p* = 5.43 × 10^−4^, Wald ratio) and the genus *Ruminococcustorquesgroup* (OR = 1.014, 95% CI = 1.005–1.023, *p* = 1.63 × 10^−3^, Wald ratio) were causally associated with colorectal cancer (Table 1, Fig. 2).

In determining more gut microbiota related to colorectal cancer in the other set of IVs (*p* < 1 × 10^−6^), we found that the order *Verrucomicrobiales*, class *Verrucomicrobiae*, and family *Verrucomicrobiaceae* (OR = 1.013, 95% CI = 1.004–1.021, *p* = 1.98 × 10^−3^, Wald ratio) were causally associated with colorectal cancer. In addition, the order *Desulfovibrionales* (OR =1.015, 95% CI = 1.005–1.025, *p* = 2.98 × 10^−3^, Wald ratio) and class *Deltaproteobacteria* (OR = 1.015, 95% CI = 1.005–1.026, *p* = 2.98 × 10^−3^, Wald ratio) were causally associated with colorectal cancer (Table 2, Fig. 4).

#### Prostate cancer

**Fig. 5:**
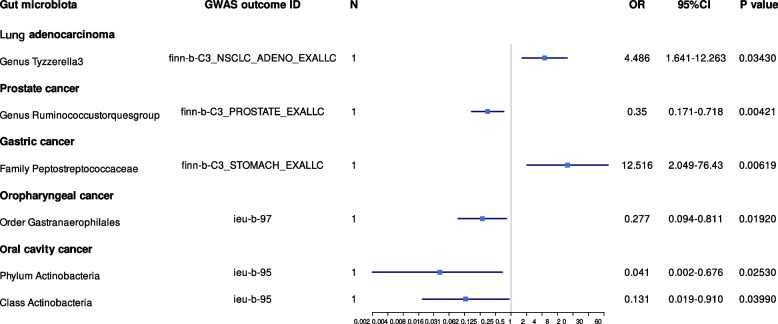
Mendelian randomisation results of causal effects between gut microbiome and cancer risk (continue) (*p* < 5 × 10^−8^)

In the set of IVs (*p* < 5 × 10^−8^), we found that the genus *Ruminococcustorquesgroup* (OR = 0.350, 95% CI = 0.171–0.718, *p* = 4.21 × 10^−3^, Wald ratio) was causally associated with prostate cancer (Table 1, Fig. 5).

In identifying more gut microbiota related to prostate cancer in the other set of IVs (*p* < 1×10^−6^), we found the class *Verrucomicrobiae*, family *Verrucomicrobiaceae*, order *Verrucomicrobiales* (OR = 0.964, 95% CI = 0.943–0.985, *p* = 8.72 × 10^−4^, Wald ratio), genus *Terrisporobacter* (OR = 0.381, 95% CI = 0.237–0.612, *p* = 6.78×10^−5^, Wald ratio), genus *Roseburia* (OR = 1.727, 95% CI = 1.276–2.337, *p* = 3.98 × 10^−4^, Wald ratio), and class *Alphaproteobacteria* (OR = 1.289, 95% CI = 1.104–1.505, *p* = 1.28 × 10^−3^, Wald ratio) to be causally associated with prostate cancer (Table 2, Fig. 4).

#### Gastric cancer

In the set of IVs (*p* < 5 × 10^−8^), we found that the family *Peptostreptococcaceae *(OR = 12.516, 95% CI = 2.049–76.43, *p* = 6.19 × 10^−3^, Wald ratio) was causally associated with gastric cancer (Table 1, Fig. 5).

However, in identifying more gut microbiota related to gastric cancer in the other set of IVs (*p* < 1 × 10^−6^), we found no genetic liability to gut microbiota that was causally associated with gastric cancer after the Bonferroni test (Table 2, Fig. 4).

#### Head and neck cancer

In the set of IVs (*p* < 5 × 10^−8^), we found that the order *Gastranaerophilales* was causally associated with oropharyngeal cancer (OR = 0.277, 95% CI = 0.094–0.811, *p* = 1.92 × 10^−2^, Wald ratio), and the phylum *Actinobacteria* (OR = 0.041, 95% CI = 0.002–0.676, *p* = 2.53 × 10^−2^, Wald ratio) and class *Actinobacteria* (OR = 0.131, 95% CI = 0.019–0.910, *p* = 3.99 × 10^−2^, Wald ratio) were causally associated with oral cavity cancer (Table 1, Fig. 5).

In identifying more gut microbiota related to head and neck cancer in the other set of IVs (*p* < 1× 10^−6^), we found that the genus *Ruminococcus1* was causally associated with head and neck cancer (OR = 1.008, 95% CI = 1.004–1.013, *p* = 1.98 × 10^−4^, Wald ratio), especially oral and oropharyngeal cancers (OR = 1.007, 95% CI = 1.003–1.011, *p* = 3.71 × 10^−4^, Wald ratio) (Table 2, Fig. 4).

#### Endometrial cancer

In the set of IVs (*p* < 5 × 10^−8^), we found that genetic liability to the gut microbiota was not causally associated with endometrial cancer, as per the Bonferroni test.

When determining more gut microbiota related to endometrial cancer in the other set of IVs (*p* < 1 × 10^−6^), we found that the class *Gammaproteobacteria *was causally associated with endometrial cancer (OR = 0.9936, 95% CI = 0.989–0.997, *p* = 8.43 × 10^−4^, Wald ratio) (Table 2, Fig. 4).

#### Ovarian cancer

In the set of IVs (*p* < 5 × 10^−8^), we did not find any genetic liability to the gut microbiota that was causally associated with ovarian cancer after the Bonferroni test.

When identifying more gut microbiota related to ovarian cancer in the other set of IVs (*p* < 1×10^-6^), we found that the genus *Ruminiclostridium 6* was causally associated with a low malignant potential in serous ovarian cancer (OR = 11.869, 95% CI = 3.178–44.327, *p* = 2.33 × 10^−4^, Wald ratio) (Table 2, Fig. 4).

### Potential causal associations between the gut microbiota and cancers

Moreover, we found some potential causal associations between the gut microbiota and cancers. Those results were found in at least two different datasets in the set of IVs (*p* < 1×10^−6^), with *p* < 0.05, but did not pass the Bonferroni test. Detailed information is provided in Additional file 3: Table S3.

The genus *Ruminococcaceae* UCG013, genus *Ruminococcaceae* NK4A214 group, genus *Oxalobacter*, genus *Holdemanella*, genus *Eubacterium eligens* group, and class *Alphaproteobacteria* were highly associated with breast cancer. The order *Selenomonadales*, genus *Turicibacter*, genus *Ruminococcus1*, genus *Ruminococcaceae* UCG014, genus *Odoribacter*, genus *Dorea*, and class *Negativicutes* were highly associated with head and neck cancer. The genus *Eubacterium coprostanoligenes* group, genus *Parasutterella*, genus *Ruminococcaceae* UCG003, and order *Lactobacillales* were highly associated with lung cancer. The family *Verrucomicrobiacea*e, class *Verrucomicrobiae*, order *Verrucomicrobiales*, genus *Terrisporobacter*, genus *Roseburia*, and genus *Akkermansia* were highly associated with prostate cancer. The genus *Adlercreutzia* was highly associated with prostate and endometrial cancer.

### Sensitivity analyses

The MR-Egger, weighted mode, simple mode, and weighted median methods yielded similar causal estimates for magnitude and direction. We found no evidence of horizontal pleiotropy for gut microbiota in cancers with *p* > 0.05 when using the MR-Egger regression intercept approach. MR-PRESSO analysis revealed no outliers in the results. In addition, the results of the Cochrane *Q* statistics showed no significant heterogeneity (*p* > 0.05).

### Bi-directional causal effects between gut microbiota and cancer risk

**Fig. 6:**
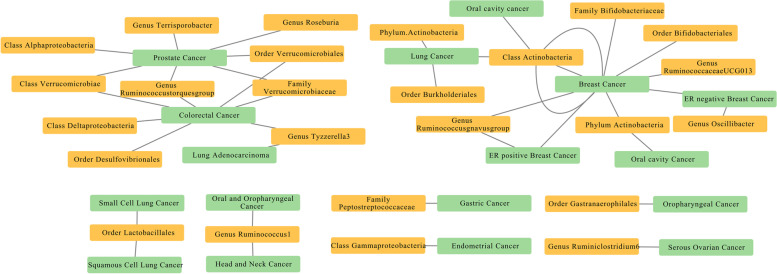
The causal relationships between gut microbiota and cancers by Mendelian randomisation analysis

To evaluate any reverse causation effects, we used cancer as exposure and gut microbiota as outcome, and 211 SNPs associated with cancers extracted from previous GWAS were used as IVs (Table 3). Based on the Bonferroni test, the significance threshold for various taxa levels was set to *p* = 6.25 × 10^−3^ (0.05/8), and we found that lung adenocarcinoma was causally associated with the genus *Tyzzerella3* (*p* = 1.02 × 10^−3^, IVW), which indicates a bi-directional causal effect between lung adenocarcinoma and the genus *Tyzzerella3*. A summary network for a better understanding of the relationship between gut microbiota and cancer is presented in Fig. 6.

**Table 3:** Bi-directional MR results of the causal effects between gut microbiome and cancer risk (*p* < 5×10^−8^)

Cancer type (exposure)	Gut microbiota (outcome)	Method	Number of SNPs	***β***	SE	***p-value***	Correct causal direction	Steiger ***p-***value
Prostate Cancer	Genus *Roseburia*	IVW	23	−0.86	0.37	1.97×10^−2^	True	1.49×10^−12^
Oropharyngeal Cancer	Genus *Turicibacter*	Wald ratio	1	79.16	33.11	1.68×10^−2^	True	8.69×10^−6^
Non-Small Cell Lung Cancer	Genus *Tyzzerella3*	Wald ratio	1	−0.16	0.06	1.14×10^−2^	True	4.48×10^−5^
Non-Small Cell Lung Cancer	Genus *Parasutterella*	Wald ratio	1	0.11	0.04	8.04×10^−3^	True	3.19×10^−7^
Non-Small Cell Lung Cancer	Genus *Tyzzerella3*	Wald ratio	1	−0.16	0.06	1.14×10^−2^	True	4.48×10^−5^
Non-Small Cell Lung Cancer	Genus *Parasutterella*	Wald ratio	1	0.12	0.04	8.03×10^−3^	True	3.19×10^−7^
Lung Cancer	Order Lactobacillales	IVW	2	−14.06	6.52	3.10×10^−3^	False	0.46
Lung Cancer	Order Lactobacillales	IVW	2	−14.42	6.51	2.67×10^−2^	False	0.48
Lung Adenocarcinoma	Genus *Tyzzerella3*	IVW	2	−0.17	0.05	1.02×10^−3^	True	1.55×10^−9^
ER+ Breast Cancer	Genus *Eubacterium eliGens Group*	IVW	7	−0.04	0.02	4.80×10^−2^	True	1.05×10^−65^
Breast Cancer	Genus *Oxalobacter*	IVW	47	0.08	0.04	2.51×10^−3^	True	6.01×10^−66^

## Discussion

To the best of our knowledge, this is the first MR study to investigate whether gut microbiota is causally associated with cancers, which we think is a longitudinal microbiome study antecedent to human cancer. Robustly associated gene variants were identified in the largest GWAS of the gut microbiota. Based on comprehensive genetic data from over 450,000 European individuals, we found genetic liability to some gut microbiota causally associated with cancers. Surprisingly, the genetic liability to the family *Bifidobacteriaceae* and order *Bifidobacteriales* was causally associated with breast cancer. We also identified some gut microbiota that might be potential risk factors for cancer. These results could have implications for public health interventions aimed at reducing cancer risk.

A growing number of studies have found a possible link between the gut microbiota selected in our study and other cancers. For instance, *Ruminococcus* plays an important role in the digestion of resistant starch [34]. However, a previous study found that *Ruminococcus gnavus* is associated with Crohn’s disease [35] and *Ruminococcus gnavus* was identified as a signature taxon for patients with hepatocellular carcinoma infected with hepatitis B and/or hepatitis C viruses [36]. The abundance of members from *Peptostreptococcaceae* was increased in patients with intrahepatic cholangiocarcinoma (ICC) compared to that in patients with hepatocellular carcinoma or liver cirrhosis and healthy individuals. Patients with vascular invasion (VI) had a greater abundance of the family *Ruminococcaceae* than did patients with ICC without VI [37].

Actinobacteria, including 15 species of *Bifidobacterium*, decreased with age, and the overall richness or number of unique *Bifidobacterium* species present in an individual steadily declined throughout their life [38]. A previous study showed that Actinobacteria was present in a relatively high proportion of breast cancer tissue samples [39]. Meanwhile, some absolute numbers of *Bifidobacterium* were significantly different according to the clinical stages of cancer [40, 41], which suggests that the microbiome may be involved in the progression of breast cancer [41]. Recent studies have reported a higher abundance of *Bifidobacterium* in the tissues of patients with colorectal adenomas [42, 43] and advanced pancreatic cancer in mice [44], whereas other studies have reported that *Bifidobacterium* correlates with an increased anti-PD-L1 therapeutic response [45, 46]. One study showed that *Bifidobacteria* might be potential pathogens [47], which indicated that although *Bifidobacterium* is generally considered beneficial, specific species and strains of *Bifidobacterium* may have varying effects on human health [48]. As shown in our results, our MR results suggested that the phylum *Actinobacteria* and class *Actinobacteria* are risk factors for breast cancer and lung cancer but are protective factors for oral cavity cancer. The family *Bifidobacteriaceae* and order *Bifidobacteriales* are also risk factors for breast cancer, while the genus *Ruminococcaceae* UCG013 is a protective factor against breast cancer. The genus *Tyzzerella3* is a risk factor for lung adenocarcinoma, but a protective factor against colorectal cancer. The genus *Ruminococcustorquesgroup* is a risk factor for colorectal cancer, but a protective factor against prostate cancer. The family *Peptostreptococcaceae* is a risk factor for gastric cancer, and the order *Gastranaerophilales* is a risk factor for oropharyngeal cancer. In summary, different species may have divergent effects on the tumour microenvironment [45].

Experimental models have suggested that gut microbiota can promote the induction and/or development of tumour formation through multiple mechanisms [49]. However, the exact mechanism by which the gut microbiota causes cancer has not been determined. Therefore, a mechanistic analysis of our results is required for further investigation.

Studies have determined whether the gut microbes are “beneficial” or “harmful” by comparing the relative abundance of gut microbiota between healthy people and patients. Engstrand and Graham suggested that the relative abundance of the dominant microbiota in the gut microbiota may not be a risk factor for cancers, but may represent a bystander effect [50] as well as a carcinogenic factor.

Many dietary components can influence cancer via targeting gut microbiota [51]. Nowadays, the prevalence of obesity is significantly increasing in developing countries such as China where people are adapting to high-fat diet [52, 53]. High-fat diet is dominated by carbohydrate and fat and lacks plant-based dietary fibre [54]. The consequence of this is that high-fat diet populations showed lower bacterial diversity compared with those of traditional rural population [55]. Obesity is correlated with excessive fat dietary intake. The positive association between obesity and cancers has been verified by several studies [56–58]. So apart from the genetic factors influencing on cancer risk, behaviour and lifestyle can also play an important role in cancer development. In the future, exploring the relationship of diet and cancer through gut microbiota may offer new insights to cancer treatment [59]. In consideration of the complex relationships between diet, gut microbiota, and cancers [60, 61], more studies and mediation MR analysis are needed to discover the association and mechanism in detail [62].

A GWAS is unlikely to explain all the heritability of complex traits [63]. As linkage disequilibrium patterns vary across ethnic groups, it is not suitable for non-European populations in the past [63, 64]. With the development of a new generation of high-density arrays and the accumulation of more sequencing data from more diverse populations, this problem may have been improved [65]. Nowadays, clinical prediction by GWASs might also prove to be especially useful in small isolated populations where deleterious variants with strong effects have increased to a high frequency [66]. A previous study revealed that variants that are significantly correlated with each other tend to be in linkage disequilibrium or even form haplotypes [67]. Although we can find causal relations between variants and disease, it is difficult to identify causal variations from multiple variants located on the same haplotype [68]. Variable penetrance and variable expressivity are the common cause for the observation where individuals carrying the same variant display highly variable symptoms [69]. A case–control analysis of autism and cancer cohorts suggested that modified penetrance of coding variants by cis-regulatory variation contributes to disease risk [70]. Although knowledge of individual’s genetic risk can improve readiness to adopt a healthier lifestyle, human behavior is complex [71, 72]. Both the environment and genes can influence disease symptoms. In addition to the separate effects of genotype and environmental factors, the effects of environmental factors on different individuals will be affected by genotype. Meanwhile, the role of genetic factors also depends on environmental influences [73, 74]. We used the MR approach to eliminate some confounders that are commonly observed in epidemiological studies. Moreover, our SNPs were strongly associated with gut microbiota and were compared with multiple cancer databases. Moreover, the reverse MR analysis and sensitivity analysis showed no pleiotropy or heterogeneity, which indicates that our results are statistically robust.

Nevertheless, our study had several limitations. First, while the majority of patients in the GWAS summary data used in our study were European, only a small number of the gut microbiota data were taken from other races, which may lead to bias estimates and affect universality. Second, the bacterial taxa were only analysed at the order or family level. If the GWASs had used more advanced shotgun metagenomic sequencing analyses, the results would be more specific and accurate. Third, due to our strict thresholds, many of the genetic liabilities of the gut microbiota were excluded at the IV selection stage, which may result in some results being missed.

Recent research proposed that future research should take an integrative approach that uses multiple omics platforms to improve understanding of the pathogenesis of disease in the context of the complex interactions between genes and the environment over time [74].

## Conclusions

In summary, we comprehensively assessed the causal association between the gut microbiota and a series of cancers. Our results suggest that there are four positive causal directions and one negative causal direction with breast cancer, three positive causal directions and one negative causal direction with lung cancer, two positive causal directions and four negative causal directions with prostate cancer, one positive causal direction with gastric cancer, one positive causal direction with ovarian cancer, one negative causal direction with endometrial cancer, six positive causal directions and one negative causal direction with colorectal cancer, and one positive causal direction and three negative causal directions with colorectal cancer. In addition, we found potential causal associations between the gut microbiota and cancer. This study may provide new insights into the mechanisms of gut microbiota-mediated cancer development.

## Supplementary Information

**Additional file 1: Table S1.** Overview of the source of cancer data.

**Additional file 2: Table S2.** F statistics of instrumental variables (IVs).

**Additional file 3: Table S3.** The potential relationships between gut microbiota and cancer risk.

## Data Availability

The summary data of Neale Lab can be downloaded from the website http://www.nealelab.is/uk-biobank/ (2022). The summary data of FINNGEN can be downloaded from the website https://www.finngen.fi/en/access_results (2022). The other datasets generated and/or analysed during the current study are publicly available and included in this published article and its supplementary information files.

## Abbreviations

BCAC: Breast Cancer Association Consortium
CRC: Colorectal cancer
FMT: Faecal microbiota transplantation
GWAS: Genome-wide association study
ILCCO: International Lung Cancer Consortium
MR: Mendelian randomisation
MRC-IEU: Medical Research Council-Integrative Epidemiology Unit
PRACTICA-L: Prostate Cancer Association Group to Investigate Cancer Associated Alterations in the Genome
SNP: Single-nucleotide polymorphism
